# Treatment of vascular graft infections: gentamicin-coated ePTFE grafts reveals strong antibacterial properties in vitro

**DOI:** 10.1007/s10856-022-06650-x

**Published:** 2022-03-10

**Authors:** Igor Lazic, Andreas Obermeier, Bettina Dietmair, Wolfgang E. Kempf, Albert Busch, Jutta Tübel, Jochen Schneider, Rüdiger von Eisenhart-Rothe, Peter Biberthaler, Rainer Burgkart, Dominik Pförringer

**Affiliations:** 1 School of Medicine, Klinikum rechts der Isar, Klinik für Orthopädie und Sportorthopädie, Technical University of Munich, Munich, Germany; 2 School of Medicine, Klinikum rechts der Isar, Klinik und Poliklinik für Vaskuläre und Endovaskuläre Chirurgie, Technical University of Munich, München, Germany; 3 School of Medicine, Klinikum rechts der Isar, Klinik und Poliklinik für Innere Medizin II, Technical University of Munich, München, Germany; 4 School of Medicine, Klinikum rechts der Isar, Klinik für Unfallchirurgie, Technische Universität München, München, Germany

## Abstract

Vascular graft infections (VGI) are severe complications in prosthetic vascular surgery with an incidence ranging from 1 to 6%. In these cases, synthetic grafts are commonly used in combination with antimicrobial agents. Expanded polytetrafluoroethylene (ePTFE) is in clinical use as a synthetic graft material and shows promising results by influencing bacterial adhesion. However, the literature on antibiotic-bound ePTFE grafts is scarce. Gentamicin is a frequently used antibiotic for local treatment of surgical site infections, but has not been evaluated as antimicrobial agent on ePTFE grafts. In this study, we examine the antimicrobial efficacy and biocompatibility of novel types of gentamicin-coated ePTFE grafts in vitro. ePTFE grafts coated with gentamicin salt formulations with covalently-bound palmitate were evaluated in two drug concentrations (GP1.75% and GP3.5%). To investigate effects from types of formulations, also suspensions of gentamicin in palmitate as well as polylactide were used at comparable levels (GS + PA and GS + R203). Antibacterial efficacies were estimated by employing a zone of inhibition, growth inhibition and bacterial adhesion assay against *Staphylococcus aureus* (SA). Cytotoxicity was determined with murine fibroblasts according to the ISO standard 10993-5. Gentamicin-coated ePTFE grafts show low bacterial adherence and strong antibacterial properties in vitro against SA. Bactericidal inhibition lasted until day 11. Highest biocompatibility was achieved using gentamicin palmitate GP1.75% coated ePTFE grafts. ePTFE grafts with gentamicin-coating are effective in vitro against SA growth and adherence. Most promising results regarding antimicrobial properties and biocompatibility were shown with chemically bounded gentamicin palmitate GP1.75% coatings.

**Figure:**
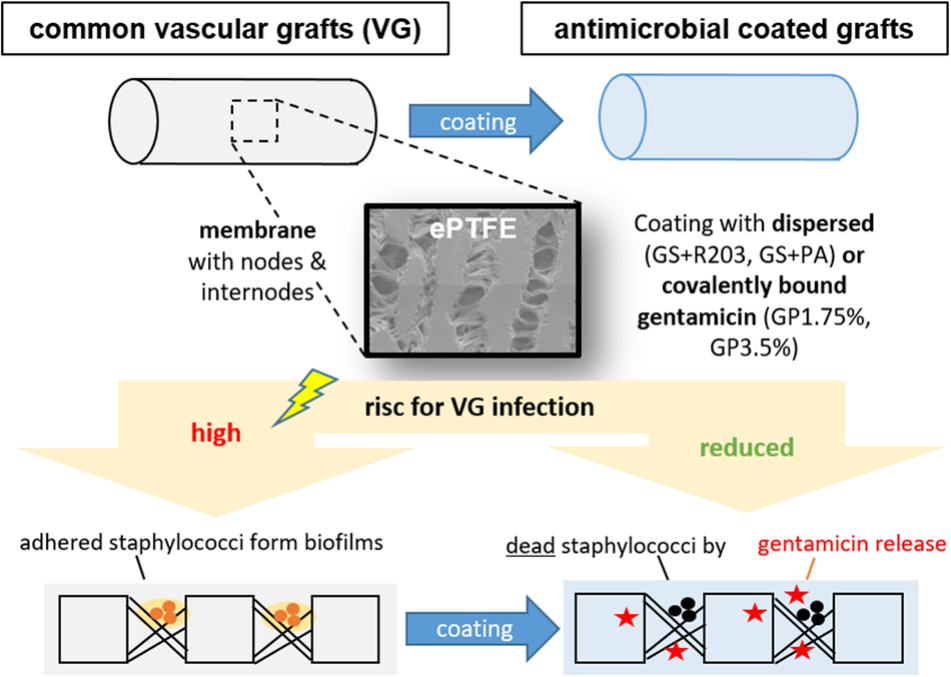
Graphical abstract

Graphical abstract

## Introduction

Vascular graft infections (VGI) represent severe complications in routinely prosthetic vascular surgery. Mortality rates between 15 and 75% have been reported with an attributed morbidity rate of 17% [1, 2]. The risk of amputation is estimated to range between 8 and 75% [2]. Incidence is ranging from 1 to 6%, depending on risk factors, such as location and material of the graft, underlying microorganisms, patient’s age, and comorbidities [3, 4]. In general, treatment implies surgical graft explantation, vascular reconstruction, and antimicrobial agents.

For this purpose, autologous or homologous grafts are available for vascular reconstruction, which can be reimplanted anatomically or extra-anatomically. In the case of VGI, anatomical reconstruction is often impractical, so that an autologous or homologous graft must be implanted extra-anatomically. However, high rates of reinfection have been reported in extra-anatomic procedures. Further complications associated with extra-anatomic procedures include occlusion, extended procedure time and risk of rupture with high rate of amputation. Seeger reported a treatment-related mortality of 19.4% and an amputation rate of 11% [1]. Thus, effective new approaches were urgently demanded [4]. In situ reconstruction, especially in case of infections, requires synthetic grafts with antimicrobial properties. In clinical practice, rifampicin-impregnated, or silver-coated vascular grafts are commonly used (Table 1).

**Table 1:**
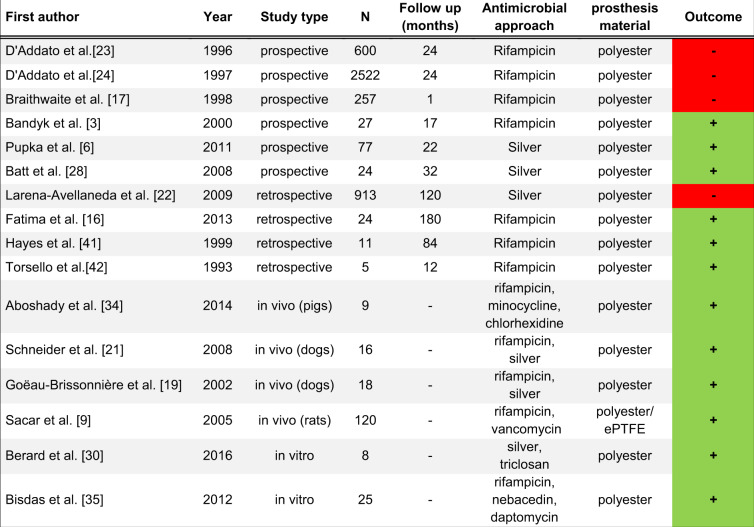
Literature overview regarding prospective, retrospective, in vitro and in vivo data on rifampicin and silver-coated vascular grafts

Studies were evaluated in terms of their outcomes (positive results (green, +), whereas negative results (red, −)

Rifampicin-impregnated grafts have been examined in clinical trials and have shown heterogeneous outcomes, whilst silver-coated grafts have shown more promising results with unclear long-term effects [5–8]. Furthermore, there is an evidence that expanded polytetrafluoroethylene (ePTFE) grafts show better antimicrobial properties than polyester grafts by influencing bacterial adhesion [9, 10]. Hence, many different therapeutic approaches are at hand, but still some uncertainty about the most effective revascularization technique remains [5, 6, 11]. The decrease of surgical site infections using gentamicin have been proven in cardiac, general and orthopedic surgery [12–14]. Gentamicin is a frequently used antibiotic for local infection treatment, as well as local infection prophylaxis. Due to its favorable thermal stability, gentamicin impregnated materials can easily be sterilized, e.g., using steam sterilization [13].

Staphylococci and gram-negative bacteria are the most common pathogens in VGI and are susceptible to gentamicin [15]. To bond antibiotics to the prosthesis in clinical practice, vascular grafts are protein-sealed and presoaked with most commonly rifampicin prior to surgery, due to its well-known effect against staphylococci [16, 17]. Only few studies investigated the effects of covalent antibiotic-coated vascular grafts, especially in the case of gentamicin [18, 19]. In the present study, we applied different gentamicin-coating formulations via dip coating for ePTFE vascular grafts and determined antimicrobial efficacy as well as biocompatibility and haemocompatibility in vitro.

## Material and methods

### Bacterial strain and cultivation

SA reference strain *Staphylococcus aureus subsp. aureus* (ATCC^®^ 49230™) was used for microbial challenge via bacterial inoculation. SA was chosen based on high incidence in VGI [15]. The bacteria were cultivated in Mueller Hinton II broth (MHB), as well as Mueller Hinton II agar (MHA), both supplied by BD, Germany. When required, an inoculum of the strain was incubated overnight under gentle shaking at 37 °C and suspended in MHB. The actual inoculum size was determined by measuring optical density at 600 nm (OD600) using a Biophotometer D30 (Eppendorf, Germany). For defined initial numbers in bacterial suspensions colony forming units (cfu) per ml were adjusted to 2 × 10^8^ cfu/ml (OD600 = 0.10). The total number of bacteria (cfu) during growth experiments was calculated via a calibration curve, representing the OD600 value dependent on the number of colony forming units per milliliter.

### ePTFE grafts

The uncoated, raw material for the prostheses was kindly provided by B.Braun, Germany. VascuGraft^®^ NEO, a standard wall ePTFE prosthesis, was used in two different geometrical shapes: for the comparative experiments circular samples with a diameter of 5 mm were punched out using a disinfected turret press (area of 39 mm²). To demonstrate reservoir depending effects cylindrical samples at diameters of 6 mm and 1 cm length were cut from tubular vascular raw prostheses material under aseptic conditions (surface area of 377 mm²).

### Preparation of vascular grafts and drug-release kinetics for characterization

A gentamicin palmitate salt formulation (GP) was supplied by Heraeus Medical GmbH, Germany. Two coating solutions were produced, one via dispersing 3.5 g gentamicin palmitate in 100 ml methanol (GP3.5%). For another low-dose gentamicin palmitate the same solution was further diluted 1:1 with methanol (GP1.75%). Gentamicin sulfate (GS) and the polylactide Resomer^®^ R203H (both from Merck, Germany) were mixed and homogenized as follows to get a gentamicin sulfate based coating solution (GS + R203): GS was immersed in 1 ml methanol, afterwards R203 was diluted in 9 ml chloroform. Then both mixtures were homogenized at 30 °C and 200 rpm for 10 min. Additionally, a dispersion of gentamicin sulfate and palmitate (GS + PA) was produced by diluting 1.14 g GS and 2.36 g PA in 100 ml methanol.

For individual coating of ePTFE grafts, samples were immersed for 30 s in the specific coating solution/suspension and dried overnight. In order to characterize antibiotic release of coated grafts, gentamicin release was measured in vitro. Therefore, gentamicin-coated ePTFE grafts were placed in Eppendorf tubes filled with 1.5 ml sterile PBS (Merck, Germany) and incubated at 37 °C with continuous shaking at 150 rpm. Elutions were taken out and ninhydrin was added at specific time intervals (0.25, 0.5, 2, 4, 8, 24, and 48 h). Ninhydrin reacts thermally with gentamicin in a dose dependent manner and generates a purple complex compound measurable by proportional absorption. Thereby gentamicin release was quantified using an UV/VIS-spectrophotometer at 405 nm (Multiskan™ GO, Thermo Fisher Scientific, Germany). Released amounts of gentamicin for different kinds of coatings were cumulated over time and results displayed in diagrams.

### Antimicrobial efficacy

Antibacterial properties of gentamicin-coated ePTFE grafts were tested against *Staphylococcus aureus* (ATCC^®^ 49230™) as a relevant bacterial pathogen model via standardized microbiological tests, described as follows.

#### Zone of inhibition assay on agar

**Fig. 1:**
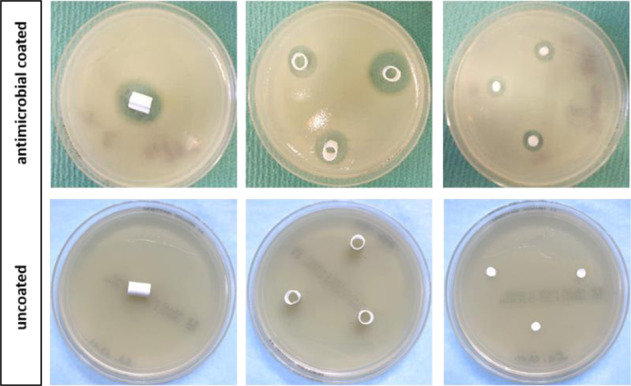
Different geometrical types of vascular graft samples for zones of inhibition testing. Left and middle: cylindrical samples on Agar, incubated with *S. aureus* overnight. Right: circular stamped samples. The lower row of pictures shows uncoated ePTFE-controls without any recognizable inhibition zone

SA was inoculated in MHB as described. 1 × 10^8^ cfu were poured into petri dishes (MHA). After 10 min of inoculation, the gentamicin-coated ePTFE grafts were placed in the middle of the petri dishes and incubated at 37 °C for 24 h. The following day, the zone of inhibition was determined using a caliper and photo documentation was performed. Afterwards, the grafts were transferred to a new petri dish with equivalent preparation. This procedure was repeated until no zone of inhibition occurred. All experiments were performed in triplicate (Fig. 1).

#### Growth inhibition in bacterial suspension

SA was inoculated in MHB as described. Bacterial suspensions were adjusted to 1.2 × 10^8^ cfu/ml (OD600 = 0.06) in 30 ml MHB using 50 ml sterile centrifugal tubes. Subsequently, gentamicin-coated ePTFE grafts were placed in the tubes and incubated under gentle shaking at 37 °C. The bacterial cell count was determined by measuring the optical density (OD600) and calculating the number of bacteria via calibration curve every hour. The experiments were performed over 8 h in triplicate.

#### Bacterial adhesion assay

SA was inoculated in MHB overnight under gentle shaking at 37 °C to maximize bacterial load. The bacterial suspension was transferred in 50 ml tubes for cylindric ePTFE graft inoculation over 2 h at 37 °C (200 rpm). The grafts were then extensively rinsed with sterile sodium chloride solution for 15 s in a shaker at 200 rpm to remove loosely adhered bacteria. These grafts were subsequently placed in sterile Eppendorf tubes containing 1 ml sodium chloride solution for 10 min sonication using a Sonorex RK31H ultrasonic bath (Bandelin, Germany). The resulting bacterial elutions were diluted by serial dilution (1:1000) and plated on MHA. The concentrations of viable adhered bacteria were determined after 24 h by counting cfu on agar. Uncoated ePTFE grafts served as a control via bacterial adhesion tests. All experiments were performed in five sets.

### Biocompatibility of gentamicin-coated grafts

Cell cytotoxicity and hemocompatibility of gentamicin-coated ePTFE grafts were used to estimate biocompatibility according to ISO standard 10993.

#### Cytotoxicity via WST-1 assay (ISO 10993-5)

Murine fibroblastic cells (L929) were grown in Dulbecco’s modified eagle medium (DMEM, Biochrom, Germany) for graft evaluation according to ISO 10993-5. Each ePTFE graft was placed in a 1.5 ml tube, covered with DMEM and incubated for 24 h at 37 °C at 300 rpm. In the same time, preculture was conducted by transferring murine fibroblasts in 96 well microplate (VWR International, Germany) and incubated at 37 °C. Initial cell sowing was 10,000 fibroblasts per well, overnight. The medium in the microplate was then removed and replaced. 200 µl of the generated ePTFE graft elutions were added. Incubation was proceeded for further 24 h. Cytotoxicity of the ePTFE grafts was estimated using the WST-1-Reagent (Roche Diagnostics, Germany) according to manufacturer specifications. Specimens were examined using ELISA reader Powerwave (Biotek, Germany) at 450 and 620 nm. The resulting absorption values were put in relation to the control to show the the percentage of sample-dependent difference in metabolic activity of the murine cells. Eight replicates were used for each sample type.

#### Hemocompatibility via ELISA and intrinsic thromboelastometry

Each ePTFE graft sample was placed in a 15 ml tube containing freshly drawn untreated human blood for 8 min contact time. After that, coagulation was stopped immediately by adding citrate (1:10) and conducting two test strains. Initially, a serum was generated to test the activated factors of blood coagulation or complement factors. Secondly, reactivated blood coagulation was tested using a ROTEM^®^ *sigma* (TEM International, Germany) according to manufacturer’s assay specifications to determine coagulation time (CT), the clot formation time (CFT) and the maximal clot firmness (MCF) after contact time with graft samples. To measure the activation of factors of the complement system a C3 ELISA Kit (AssayMax, USA) and for coagulation factors a TAT Elisa Kit (Abcam, USA) were performed according to manufacturer specifications. Freshly drawn human donor blood as well as uncoated ePTFE graft served as controls.

### Scanning electron microscope (SEM)

For SEM pictures to investigate surface adhered bacteria, gentamicin-coated vascular grafts were inoculated as described for growth experiments in 30 ml SA suspensions. Incubation lasted for 2 as well as 24 h at 37 °C at 200 rpm. The grafts were then extracted from the tubes, washed with sterile water and fixed with paraformaldehyde for 15 min, washed again with sterile water and examined using a scanning electron microscope S3500 N (Hitachi High Technology Europe GmbH, Germany).

### Statistics

Mean values and standard deviations were calculated from at least five independent measurements. Two-tailed Student’s *t* test was performed for testing on equality of data sets at significance levels *p < 0.05; **p < 0.01; ***p < 0.001. The distribution of data was checked for each group and taken into account during student’s *t* test. GraphPad Prism 5.0 (GraphPad^®^ Software, La Jolla, CA, USA) was used for data evaluation and visualization of the resulting graphs.

## Results

### Preparation of vascular grafts and drug-release kinetics for characterization

**Fig. 2:**
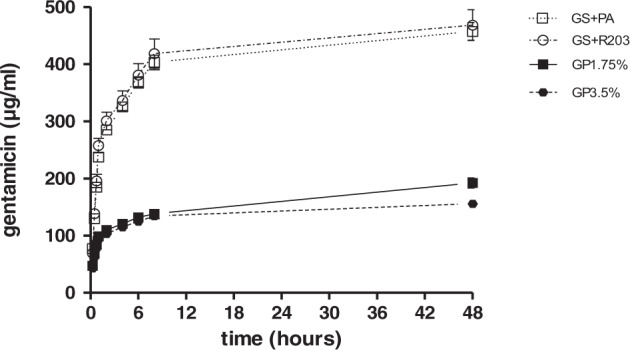
Gentamicin-release in PBS solution over 48 h for vascular graft coatings using gentamicin in various formulations

The loading mass of the coated ePTFE grafts was analyzed as shown in Table 2. The preparation of the grafts could be validly reproduced. Among the differently coated ePTFE grafts the loading masses varied. GS + R203 was the heaviest coating, followed by GS + PA. The weight of both GP1.75% and GP3.5% were comparably lower. The load of gentamicin per surface area of the ePTFE grafts exhibited proportional effects: GS + R203 showed the densest gentamicin loading per surface area, followed by GS + PA, GP3.5, and GP1.75%, in that order. Thereby, the proportion of gentamicin per surface area of GP1.75% contained half the proportion of gentamicin per surface area of GP3.5%. To characterize antibiotic release of the coated ePTFE grafts, gentamicin release was measured in vitro (Fig. 2). The release of the gentamicin reaches an approximately steady level after the first 48 h. GS + R203 and GS + PA reach comparable amounts of gentamicin until first 8 h at 418 µg/ml and 403 µg/ml, respectively. Whereas the palmitate containing coatings GP3.5% and GP1.75% released similar amounts of gentamicin for 8 h on a lower extend, reaching 134 and 137 µg/ml.

**Table 2:** Gentamicin loading among the different coating types on cylindrical ePTFE graft samples

Coating type	Coated ePTFE graft	Coated mass	Proportion gentamicin	
	Ø Weight (µg)	Ø Weight (µg)	Ø Weight (µg)	Per area (ng/mm^2^)
GP1.75%	990 ± 14	13	3.4	9
GP3.5%	985 ± 84	40	8.1	21
GS + R203	1343 ± 11	113	67.7	180
GS + PA	1228 ± 33	15	12.3	33

### Antimicrobial efficacy

#### Zones of inhibition on agar

**Fig. 3:**
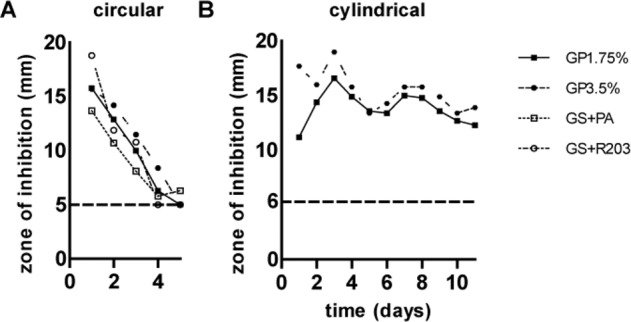
Zones of inhibition against SA using circular and cylindrical samples of gentamicin-coated ePTFE grafts. **A** circular coated samples with GP1.75%, GP3.5%, GS + PA and GS + R203. **B** cylindrical samples of gentamicin-coated ePTFE grafts with GP1.75% and GP3.5%

Long-term SA inhibition in diffusion tests regarding circular probes are displayed in Fig. 3A. All different coatings sustained a zone of inhibition against SA until day 5. By day 6, the bacterial colonization was the same for the gentamicin-coated as for the uncoated grafts. No significant dose dependent difference occurred regarding GP1.75% and GP3.5%. The largest zones of inhibition were measured on day 1 for all coatings. GP3.5% and GS + R203 showed the greatest zone of inhibition by day 1, whereby it should be noted that the GS + R203 had the greatest fluctuations of growth inhibition over time. The smallest zone of inhibition was noted with GS + PA. To examine dose and drug reservoir depending effects on efficacy and duration of antimicrobial efficacy, a diffusion test was performed with different geometrical shapes, according to different amounts of gentamicin. Figure 3B shows the long-term SA inhibition of grafts coated in the form of cylinders. Hence, the zone of inhibition lasted until day 11 using cylinders having the highest amount of gentamicin, biggest surface and thus highest reservoir resulting in a longer efficacy. Again, no significant difference between GP1.75% and GP3.5% could be demonstrated.

#### Growth inhibition in bacterial suspension

**Fig. 4:**
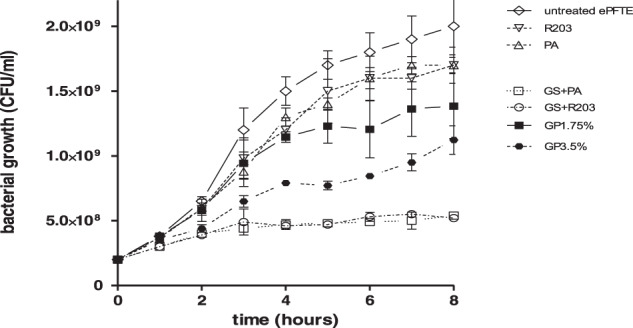
SA growth inhibition in bacterial suspension with circular probes of untreated, non-gentamicin, and gentamicin-coated ePTFE grafts

To analyze the various coatings with gentamicin, further bacterial growth tests using SA suspensions were performed. Preliminary results showed the highest release of gentamicin within the first 8 h. The results of growth inhibition using circular probes in bacterial suspension are demonstrated in Fig. 4. Bacterial growth was stronger in the uncoated ePTFE graft in comparison to ePTFE grafts coated with PA or R203. However, all coatings containing gentamicin resisted colonization more effectively.

GP1.75% and GP3.5% show stronger antimicrobial properties than the untreated ePTFE grafts, PA and R203. No significant differences between GP1.75% and GP3.5% were observed in the first 8 h. The strongest antimicrobial effect was seen in GS + PA and GS + R203, which contain the highest levels of antibiotics in the experiment.

#### Bacterial adhesion assay

**Fig. 5:**
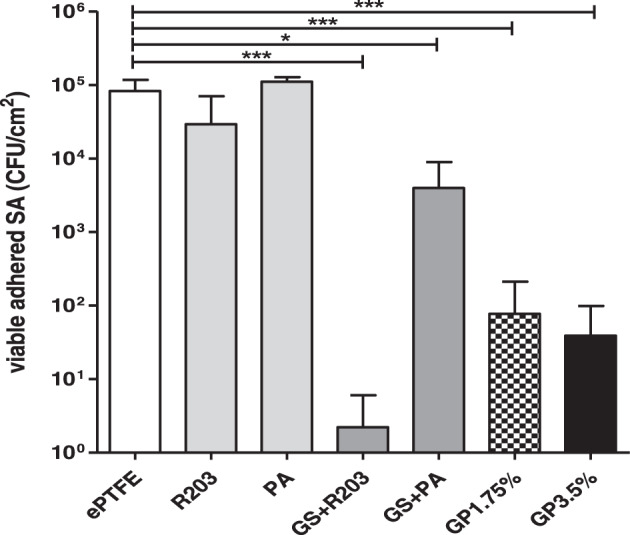
Numbers of adhered SA on untreated, non-gentamicin and gentamicin-coated ePTFE grafts

Numbers of viable adhered bacteria on the different uncoated and antimicrobial coated vascular grafts are shown in Fig. 5. Pure palmitate coatings showed increased adherence at 111 × 10^3^ cfu/cm^2^ (−0.1 Log) in comparison to the untreated ePTFE graft. Polylactide coatings without antimicrobial drug (R203) led to a significant reduction towards 29 × 10^3^ cfu/cm^2^ (0.5 Log). The gentamicin-coated grafts showed highly reduced bacterial adherences, 4 × 10^3^ cfu/cm^2^ (1.3 Log) in GS + PA, 39 cfu/cm^2^ in GP3.75% (3.3 Log) and 77 cfu/cm^2^ (3.0 Log) in GP1.75%, respectively. Only 2 cfu/cm^2^ could be detected for GS + R203 (4.6 Log).

### Biocompatibility of gentamicin-coated grafts

#### Cytotoxicity via WST-1 assay (ISO 10993-5)

**Fig. 6:**
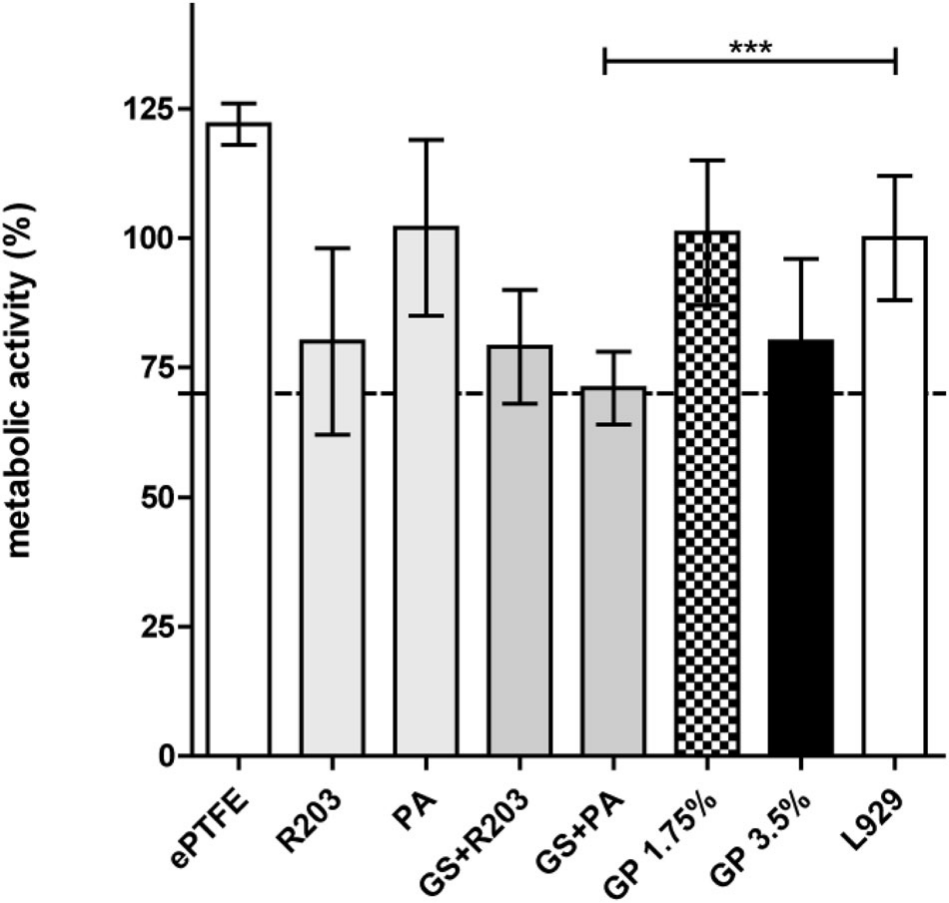
Metabolic activity of murine fibroblasts (L929) using a WST-1 assay. L929 were challenged with the DMEM elution of untreated, non-gentamicin and gentamicin-coated ePTFE grafts respectively (*n* = 7). Dotted line indicates the limit of tolerated loss of activity at 70% according to the ISO 10993-5 standard for medical devices

The metabolic activity of mammalian cells was measured by WST-1 assay as shown in Fig. 6. The cells’ metabolic activity was accelerated to 122% on the untreated ePTFE grafts. Coatings without active substances polylactide coatings (R203) caused a little decrease of 20%, whereas PA kept unaltered activity at 102%. Antibiotic-coatings using dispersed Gentamicin, GS + R203, decreased cell activity to 79% and GS + PA to 71% presumably because of its higher gentamicin release. Compared to covalently-bound Gentamicin coatings with PA, GP3.5% resulted in a decrease to 80%, whereas GP1.75% led to no negative change in cell activity referred to L929 growth, due to the lower concentration.

#### Hemocompatibility via ELISA and intrinsic thromboelastometry

To analyze hemocompatibility and activation of the complement system, intrinsic thromboelastometry and ELISA assay of C3a and TAT were performed. The results are summarized in Table 3. No significant differences using the different types of coatings could be observed regarding activation of the complement system via C3a (additional data shown in Appendix).

**Table 3:** Coagulation properties of gentamicin-coated ePTFE grafts and the control groups (human blood, untreated ePTFE graft, and ePTFE grafts coated only with PA respectively R203)

Sample type	CT (sec.)	MCF (mm)	CFT (sec.)
Control	174 ± 2	52 ± 2	109 ± 15
ePTFE	147 ± 5	52 ± 2	110 ± 10
R203	148 ± 16	55 ± 1	94 ± 6
PA	131 ± 11	46 ± 10	164 ± 68
GS + R203	208 ± 9	56 ± 1	87 ± 10
GS + PA	157 ± 7	56 ± 1	88 ± 6
GP1.75%	165 ± 22	53 ± 2	110 ± 22
GP3.5%	178 ± 13	53 ± 1	96 ± 6

Coagulation time, clot formation time, and mean clot firmness measured via intrinsic thromboelastometry are shown in seconds and millimeters, respectively

### Scanning electron microscope

**Fig. 7:**
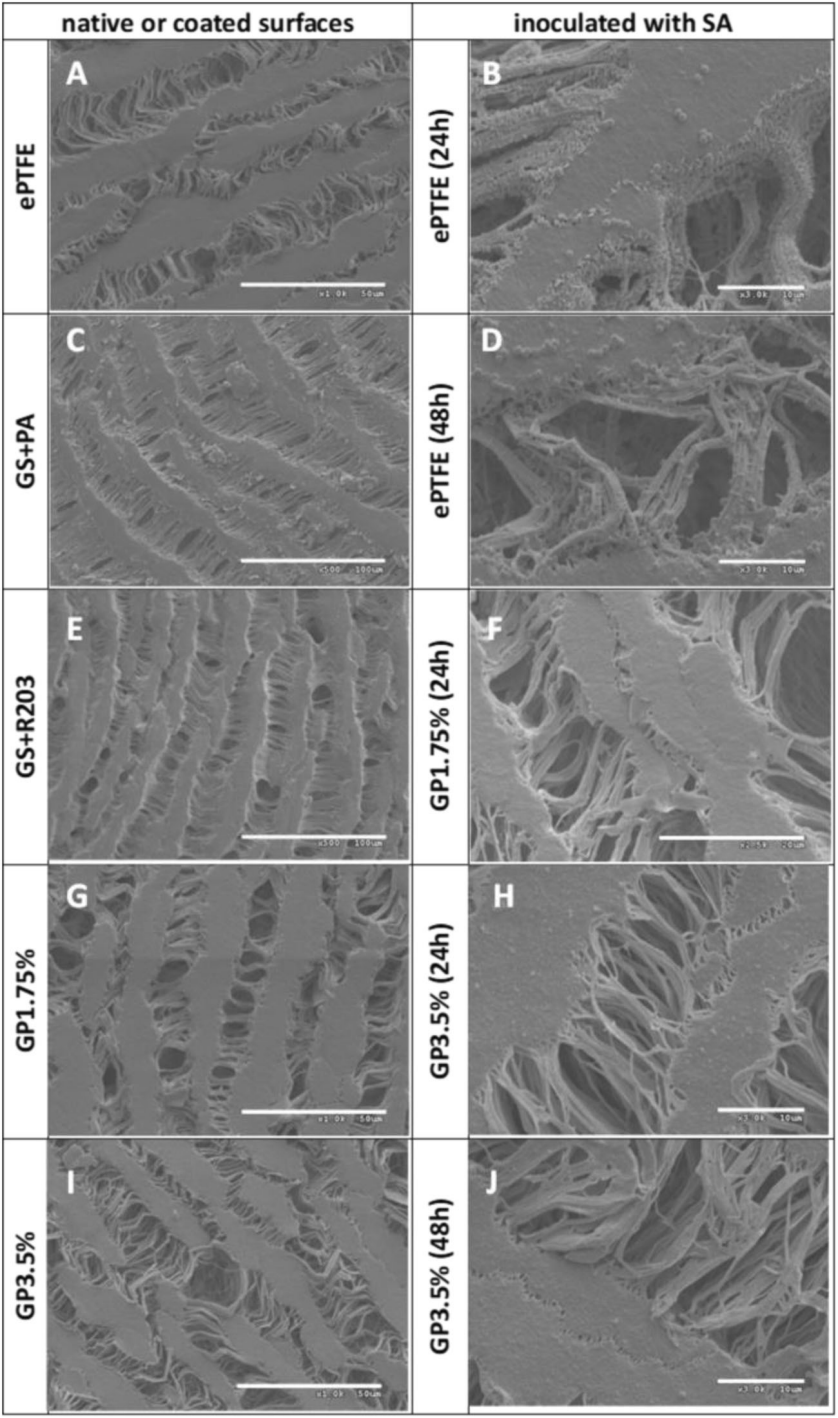
SEM pictures of uncoated and various gentamicin-coated ePTFE grafts (left) and with SA incubated grafts (right) exemplarily after 24 and 48 h. **A** Non-coated ePTFE graft (×1000); **B**, **D** ePTFE graft (×3000) after SA inoculation time; **C**, **E**, **G**, **I** diverse coated surfaces for GS + PA, GS + R203 and GP variants; **F**, **H**, **J** show GP coating variants after 24 and 48 h of SA exposure at 37 °C indicating highly reduced numbers of adhered SA compared to uncoated ePTFE graft (**B** + **D**)

The untreated ePTFE graft after SA inoculation demonstrates bacterial growth, especially in the internodes of the common PTFE structure. Gentamicin coatings on ePTFE graft surfaces protect effectively against these kinds of bacterial colonization at 48 h inoculation. In detail, isolated staphylococci can be detected on the prosthesis surface after 2 h inoculation and cannot be considered for quantitative comparison (Fig. 7A–J). Regarding the uncoated graft and antimicrobial coated surfaces themselves: The common surface of an untreated ePTFE prosthesis can be seen without (Fig. 7A) and with bacterial colonization (Fig. 7B, D). In comparison, coatings with the higher concentrated GP3.75% (Fig. 7G) indicate finely contoured surfaces. In contrast, the surface coated with GS + PA (Fig. 7C) shows the coarse particle-like structure of dispersed gentamicin in palmitate. Moreover, the use of polylactide GS + R203 (Fig. 7D) leads to a “lacquering” of the fibrillary internodes. Bacterial colonization of the respective grafts is demonstrated in Fig. 7F, H, J. Longer inoculations of 48 h show significantly reduced bacterial counts for the differently coated prosthetic surfaces. It should be pointed out that untreated ePTFE grafts show numerous bacterial adhesions (Fig. 7B, D), compared to the GP3.75% coatings (Fig. 7J) shown here exemplarily for the antimicrobial coatings.

## Discussion

VGI can pose a life-threating complication in vascular surgery. A number of treatment options, including different revascularization techniques and antimicrobial agents are at hand, but results seem relatively unsatisfying. Infrarenal aortic graft infections were associated with a perioperative mortality rate at 12–27% [8]. Staphylococci and gram-negative bacteria are the prevalent pathogens in VGI. Hence, administered antibiotics should cover these pathogens. Gentamicin is an aminoglycoside with well-known antimicrobial effects in general, cardiac and orthopedic surgery [12–14]. Using impregnated implants leads to high local gentamicin concentrations, whereas serum concentration correspondingly stays low. Thus, toxicity and side effects, like kidney damage and hearing loss, can be avoided. In addition, the risk of antibiotic resistance due to insufficient prolonged systemic antibiotic administration is lowered [12]. Both staphylococci and gram-negative bacteria are susceptible to gentamicin [12–14]. Apparent on formal validation, treatment with aminoglycosides is beneficial in the VGI setting. In a clinical trial, Szczot et al. demonstrated that early gentamicin administration was associated with a favorable outcome for patients with VGI in the intensive care unit [20]. Furthermore, Almeida et al. demonstrated in a pilot study the efficacy of local application of gentamicin using impregnated collagen implants in ischemic patients submitted to femoropopliteal PTFE bypass. Surgical site infections and in-hospital days in the treatment group were reduced. Noteworthy, in this case local gentamicin was released by a subfascial collagen implant, not a gentamicin-coated vascular graft.

In situ reconstruction has been demonstrated as a safe option to extra-anatomic reconstruction with lower perioperative mortality and lower amputation rate [5]. It is evident that the implantation of any synthetic material carries the risk of bacterial infection with formation of a biofilm, an extracellular polymeric matrix protecting the pathogens from host immune defense and antibiotics [21]. In situ reconstruction therefore uses synthetic grafts with antimicrobial properties. In clinical practice, the predominant antimicrobial agents in VGI treatment are rifampicin and silver [5, 22].

Rifampicin-coated grafts have shown heterogeneous results in graft reconstruction. O’Connor et al. systemically reviewed rifampicin-coated grafts in the treatment of aortic graft infection. Their results showed lower amputation rate, mortality and conduit failures in comparison to extra-anatomic procedures, homo- and allografts [5]. Goëau-Brissonnière et al. demonstrated in a dog model that rifampicin-coated grafts show significantly higher resistance to bacterial infection than silver-coated grafts, although it remains uncertain if these results are applicable in a human scenario. In contrast, Earnshaw demonstrated no significant difference between uncoated and rifampicin-coated polyester grafts for extra-anatomic reconstruction [11]. Similarly, D’Addato et al. could not state a significant difference in two prospective multicenter clinical trials concerning the prevention of VGI by rifampicin presoaked polyester grafts in 600 and 2522 patients, respectively [23, 24]. However, a lack of activity against Gram-negative bacteria and MRSA should be reason to consider other antibiotics [7]. Additionally, the severe reduction of antibiotic properties after ~1 week [7, 8] and the high cytotoxicity of rifampicin against vascular cells need to be taken into consideration for therapeutic decisions [25].

Overall, mortality and reinfection rate in vascular reconstruction with rifampicin-coated grafts are still high [5, 7]. Furthermore, in approximately 30% of cases, microbial organisms isolated from infected grafts are resistant to rifampicin [26]. Advantages of silver-coated vascular grafts are the wide antimicrobial activity including MRSA and the lack of resistance development [7]. The antimicrobial potential of silver in medical use has been widely reported [6, 27]. Silver interacts with bacterial peptides and thereby inhibits colony formation. It has been demonstrated that silver-coated polyester grafts can be as effective as arterial allograft. Bacterial resistance to silver is low, whereas resistance to antibiotics emerges fast [6]. Unfortunately, late recurrent infection (11%) represents the essential drawback of silver-coated grafts [28, 7]. More recently, vascular grafts combining silver and triclosan have shown promising short-term antimicrobial activity in vitro [29, 30]. However, literature concerning the combination of silver and triclosan is still scarce. So far, it is unclear whether rifampicin- or silver-coated grafts are favorable.

Various antibiotics have been evaluated in vitro in the setting of VGI [31, 32, 10, 33–35]. However, the effects of gentamicin-coated vascular grafts are not well defined yet [18, 19, 13]. One other study at hand evaluated the in vitro effect of gentamicin-coated vascular grafts, showing that covalently immobilized gentamicin on polyester grafts has superior antibacterial properties in comparison to silver-coated polyester grafts [18].

In this study, we evaluated different types of gentamicin coatings on ePTFE grafts and analyzed their antibacterial effect after exposure to SA. To our knowledge, no other gentamicin coatings for ePTFE grafts have been successfully tested yet.

We could demonstrate that our preparation techniques lead to reproducible coatings and steady drug-release kinetics. However, the coatings showed differences in the total weight among each other and therefore the total amount of released antibiotics varied notably. GS + R203 had the highest gentamicin proportion, although most of its mass consisted of the Resomer^®^. Remarkably, GS + R203 still showed the 18-fold amount of gentamicin compared to GP1.75%. Accordingly, GS + R203 showed the highest release of gentamicin. Furthermore, as expected, higher absolute amounts of coated gentamicin showed prolonged growth inhibition. Therefore, GS + R203 showed the strongest and longest lasting antibacterial effect in the presented experiments. The described fluctuation of antibacterial activity and the strong cytotoxicity of GS + R203 in the biocompatibility testing should be seen in this context.

GS + PA was able to release similarly high levels of gentamicin as GS + R203 despite a significantly lower proportion of gentamicin. This indicates that the dispersion with GS + GP can enable a high drug-release already in static conditions in vitro. Even though gentamicin is covalently bound to palmitate, gentamicin is not covalently bound to ePTFE. Gentamicin palmitate is merely adsorbed onto the surface of ePTFE. Teflon is known to be both hydrophobic and oleophobic. Rapid release can therefore be assumed if the coated ePTFE is implanted in a surgical setting.

Interestingly, GP3.5% showed twice the amount of gentamicin in its coating compared to GP1.75%, but the antibiotic release of the lower dosage GP1.75% shows only minor differences compared to GP3.5% in regard to antimicrobial effects. Although there was a dose dependent effect for GP1.75% and GP3.5% with circular probes in SA suspension, there was no consistency of the results in the experiments overall. Hence, we could not demonstrate a significantly stronger antibacterial effect using the GP3.5% coating in comparison to GP1.75%, although we validated a considerable decrease in cell viability with GP3.5%. These results indicate that a coating with GP1.75% has a higher benefit/risk ratio in this setting. The dispersion GS + PA showed a comparable antibacterial activity. Bacterial adherence was low in comparison to the non-gentamicin coatings, but still 3-fold higher than in the GP-coatings, indicating that the chemically bound GP somehow has a superior antibacterial effect. An overview to evaluate the properties of the different types of ePTFE coated graft samples using gentamicin is demonstrated in Table 4.

**Table 4:** Evaluation of the different types of gentamicin-coated ePTFE grafts

Type of graft	Antibiotic concentration	Antimicrobial efficacy		Growth inhibition	Resistance to bacterial adhesion	Cytotoxicity	Hemocompatibility
	Gentamicin (ng/mm^2^)	Duration (d)	Zone of inhibition (mm)	Log reduction of bacteria in suspension	Log reduction of adhered bacteria	Reduction of metabolic activity (%)	Reduction of coagulation time (%)
GS + R203	+++	+++	+++	+++	+++	+++	−
GS + PA	++	++	+	+++	+	+++	−
GP1.75%	+	++	+++	++	++	−	−
GP3.5%	+	+++	+++	++	++	++	−

Commercially available vascular grafts contain a protein sealing, preoperatively loaded with rifampicin [16]. An advantage of covalent binding of gentamicin to the graft is that soaking is redundant and the graft may be used immediately. It can be assumed that standard processing of the grafts with covalently-bound gentamicin will result in a more homogenous antibiotic loading compared to the preoperative soaking in rifampicin, which is currently common practice. Furthermore, ePTFE grafts have been considered the more favorable option for in situ replacement, especially in case of infection [36]. A highly hydrophobic surface and nonporous structure result into lower bacterial adhesion as compared to polyester grafts [9]. Here, we demonstrated that gentamicin-coated ePTFE grafts show least amounts of bacterial adherence. The most powerful effect was seen in GS-R203 with no bacterial adherence at all. Bacterial adherence is a crucial step in biofilm formation and thus in VGI. The relative resistance to bacterial adhesion associated to the ePTFE graft poses a substantial advantage in in situ replacement techniques. A higher bacterial affinity to polyester in comparison to ePTFE has already been described [37]. Different protein exposure on the grafts can mediate bacterial adhesion, which differs in polyester and ePTFE. Polyester graft fibers consist of woven multifilaments, resulting in a large available surface area, whereas the ePTFE grafts are relatively nonporous. Davidovic et al. demonstrated high levels of protein exposure on Dacron^®^ grafts and the lowest on ePTFE. In this study, we could demonstrate that any gentamicin-coating further diminished bacterial adherence on the ePTFE grafts. Moreover, scanning electron microscope examination of the grafts after inoculation with SA revealed, that bacteria could be detected in the internodes of the graft. Coating with gentamicin seems to fill these spaces and thus reduces the bacterial growth by reducing the surface area, whilst polyester fibers still pose a greater surface even after gentamicin coating. Further research should examine the bacterial adherence on gentamicin-coated ePTFE grafts in comparison to gentamicin-coated polyester grafts, to confirm these findings. Regarding hemocompatibility of the grafts, the gentamicin coatings slightly altered the coagulation time. Again, GS + R203 showed the strongest effect by extending CT in comparison to the uncoated graft. Similarly, GP3.5% marginally extended CT, whereas GP1.75% showed no significant difference to the uncoated graft. Regarding activation of the complement system no significant differences could be observed. These results indicate that GP1.75% and GP3.5% coatings are as hemocompatible as the commercially available ePTFE graft used in these experiments (Table 5).

**Table 5:** Rating levels used for the comparative evaluation of different grafts

Properties of different grafts		Rating levels			
		−	+	++	+++
Antibiotic concentration	Gentamicin (ng/mm^2^)	−	<30	>30	>100
Antimicrobial efficacy	duration (d)	−	>1	>3	>5
	Zone of inhibition (mm)	5	>5	>10	>15
Growth inhibition	Log reduction of bacteria in suspension	−	>0.3	>0.6	>0.9
Resistance to bacterial adhesion	Log reduction of adhered bacteria	<1	>1	>2	>4
Cytotoxicity	Reduction of metabolic activity (%)	0	>10	>20	>30
Hemocompatibility	Reduction of coagulation time (%)	0	>10	>20	>30

This study exhibits several limitations. We demonstrated an effective inhibition of bacterial growth using gentamicin-coated ePTFE grafts up to 11 days. A major concern in reconstruction with synthetic grafts is the quick elution of the coated antimicrobial agents, thus leaving the graft susceptible to late infection.

The European Society for Vascular Surgery has recently stated that there is no consensus on the optimal length of antimicrobial therapy for VGI, at all. A strong recommendation was given to cover the first 24 h in the purpose of antibiotic prophylaxis. The duration of the further intravenous, oral and/or local antibiotic therapy is still a matter of discussion and may vary from 2 weeks to 1 year postoperatively [38]. Data regarding the antibiotic release kinetics of both clinically established grafts and gentamicin-coated ePTFE grafts is scarce. In this study, we aimed to evaluate different types of gentamicin coatings. By increasing the dose, the duration of the inhibition time was effectively prolonged. However, evaluating the longest lasting antimicrobial effect was just a minor aspect leaving the favorable coating dose concerning long-term effects and toxicity yet to be determined. Furthermore, in the present study, we only examined the effects of GP against SA due to its high incidence in VGI. The most common pathogen in VGI are SA and *S. epidermidis*. Gram-negative bacteria like *Escherichia coli* and *Pseudomonas aeruginosa* have been described, as well [39]. Gentamicin is well known for its effect in gram-negative bacteria. Aminoglycosides are generally used to treat severe infections with staphylococci and their effects have been confirmed [15]. Although clinical effects of gentamicin in gram-negative bacteria have been widely reported, further investigation of the effect of GP in other pathogens is necessary—especially in polymicrobial infections, which often occur in VGI [13]. Furthermore, the effects of the gentamicin-coated ePFTE grafts have not been evaluated in vivo in the scope of this study. A thorough investigation of gentamicin-coated grafts in vivo is intended. However, our laboratory protocol and ethics committee requires the submission of peer-reviewed publications for such costly and elaborate animal models evaluating implant infections. Hence, the in vivo analysis will follow in a subsequent study. Another limitation of this study is that the effects of gentamicin coatings were investigated without comparing them to clinically applied grafts in VGI or other promising antimicrobial techniques in implant surgery. We elaborated that it is uncertain whether rifampicin- or silver-coated grafts are favorable. Moreover, various other techniques of antimicrobial coatings show promising results i.e., with octenidine, triclosan, chlorhexidine, or adaptive release of metal ions [29, 40]. Hence, extensive comparative studies in vivo are necessary. We therefore assume that currently an final interpretation of the release kinetics is not feasible, neither in the scope of the in vitro nor in vivo studies. A clinically significant effect of gentamicin-coated ePFTE grafts might be conceivable, even with the fast release seen in the current results.

## Conclusions

In conclusion, we demonstrated several options of gentamicin coating on ePTFE grafts, of which GP1.75% showed the most promising results concerning antibacterial effects against SA and cytotoxicity in vitro. We demonstrated that covalently-bound gentamicin had a superior effect in comparison to the other coatings. Chemically bound gentamicin on the graft may therefore represent a promising alternative to the presoaking of polyester grafts, which is clinically applied so far. Moreover, gentamicin coating did not alternate cell viability and coagulation properties significantly, indicating low cytotoxicity and good hemocompatibility of the modified grafts. Furthermore, we were able to demonstrate further reduction of bacterial adherence through the gentamicin coating on the already favorable ePTFE grafts in this respect. Additional studies are crucial to confirm these results, especially in comparison to the rifampicin-coated and silver-coated polyester grafts, which are established in clinical practice.

## Data Availability

Data are available from the authors upon reasonable request and with explicit permission of the participants.
